# Physical training interventions for children and teenagers affected by acute lymphoblastic leukemia and related treatment impairments

**DOI:** 10.18632/oncotarget.24762

**Published:** 2018-03-30

**Authors:** Carolina Simioni, Giorgio Zauli, Alberto M. Martelli, Marco Vitale, Simona Ultimo, Daniela Milani, Luca M. Neri

**Affiliations:** ^1^ Department of Morphology, Surgery and Experimental Medicine, University of Ferrara, Ferrara, Italy; ^2^ Department of Biomedical and Neuromotor Sciences, University of Bologna, Bologna, Italy; ^3^ Department of Medicine and Surgery, University of Parma, Parma, Italy; ^4^ CoreLab, Azienda Ospedaliero-Universitaria di Parma, Parma, Italy

**Keywords:** acute lymphoblastic leukemia (ALL), cancer treatments, childhood, physical assessment tests, physical exercise

## Abstract

A decreased physical fitness has been reported in patients and survivors of acute lymphoblastic leukemia (ALL). This is influenced by the negative effects of the disease and by the treatments of childhood cancer.

In the past, children were advised to recover in bed, and to take as much relax as possible. Nowadays, it is considered that too much immobility may result in a further decrease of physical fitness and functioning. Exercise training for ALL children has frequently been reported to improve physical fitness and the well-being of the children, since it prevents the negative effects of a sedentary life-style, such as obesity and a poor skeletal health. In recent years, different studies and protocols on this subject has become available for children and young adults with cancer, both during and after treatment.

The efficacy of recent physical exercise training interventions, that act on several ALL impairments in children such as skeletal, musculoskeletal, neuromuscular, cardiopulmonary and cardiovascular systems, fatigue, body balance disorders and metabolism alterations have been examined.

These side effects might be prevented or significantly reduced by introducing a physical exercise program during or shortly after cancer treatment. Several interventions are discussed and presented for each impairment, reducing their level caused by the disease and thus suggesting the importance of physical training activity in ameliorating the children quality of life.

## INTRODUCTION

Acute lymphoblastic leukemia (ALL) is an aggressive neoplasia characterized by the uncontrolled proliferation of aberrant lymphocytes, and represents the most common childhood cancer [1–3]. Over years, several efforts have been made for the best treatment approach, such as combination of chemotherapy and radiotherapy [4, 5]. These therapies, however, may impair the whole child health status and quality of life through different side effects which include osteopenia, muscle atrophy, cardiovascular and cardiopulmonary problems or metabolism alterations [6–9]. Pharmacological therapies such as dexamethasone and prednisone treatments induce significant changes in bone morphology and affect both the musculoskeletal and neuromuscular systems [10]. Physical therapy and physical activity are an increasing interest of the pediatric oncology population [11–15]. The number of exercise interventions, as well as physical therapy programs are in constant development, and there is evidence that they can decrease fatigue and other critical impairments which usually develop both during the course of the disease and during and after treatments. The ideal modality associated with the intensity and duration of the intervention need to be personalized to apply precision medicine and obtain the best benefits [16–18]. The purpose of this review is to assess the relevance of physical activity in the most frequent impairments found in patients with ALL. In this review we aim to focus on skeletal bone loss and neuromuscular impairments, cardiovascular and cardiopulmonary disorders, one psycho-physical aspect, cancer-related fatigue, balance impairment, obesity and metabolism disorders. For each impairment we report some of the recent advances in physical training programs for childhood ALL patients, tests for assessment when available, the most recent exercise intervention protocols developed and the benefits that these programs may give the different impairments.

### Skeletal bone loss impairment

Children with ALL are often affected by bone and musculoskeletal pain, especially in the extremities, dorsal spine and pelvis, and gait disturbances [19–23]. Indeed, acute lymphoblastic leukemia causes severe joint lesions and a general bone weakening, with a consequent loss of bone mineral mass and perturbation of mineral homeostasis. All these phenomena are due to the common pathology known as decreased Bone Mineral Density (BMD). Reduced bone turnover has been reported at diagnosis and during the treatment of ALL [24, 25]. Soluble products of malignant cells like lymphotoxin, interleukin-1 (IL-1), interleukin-8 (IL-8), tumor necrosis factor (TNF) or osteoclast activating factor (OAF) may be the cause of and contribute to bone demineralization [26, 27]. Treatments with corticosteroid and methotrexate decrease bone development and increase bone resorption with consequent net loss of bone mineral [28, 29]. Glucocorticoid therapy, in general, is reported to induce gradual lipid accumulation within osteocytes and osteocyte apoptosis, thereby suppressing osteoblastic differentiation [30, 31]. These abnormalities increase in frequency and seriousness as treatments progress, and begin to resolve after end of therapy, suggesting that treatments of ALL play a role in the onset of osteopenia.

The bone loss could be accelerated by hyperthyroidism and hyperparathyroidism, by cortisol and related steroids as well as estrogen and testosterone deficiencies [19]. Moreover, some chemotherapeutic agents such as platinum cause nephrotoxicity, resulting in impairments of calcium balance through vitamin D reduction.

Magnetic resonance imaging (MRI) is one of the most sensitive method to identify and quantify osteonecrosis in children and adults and is usually performed several months after the initiation of treatment or routinely at completion of treatment. The Association Research Circulation Osseous (ARCO) proposed a classification of the size of the necrotic area, in which osteonecrosis is classified into 4 different stages ranging from normal diagnostic images to evident changes in the femoral articular surface [10, 32, 33].

Exercise programs or physical activity interventions have been studied to prevent skeletal bone impairments for children with newly diagnosed ALL. Two most relevant methods to estimate functional mobility in ALL children are the timed up and down stairs (TUDS) and the Timed Up and Go (TUG) tests.

TUDS measures the time (in seconds) needed to ascend and descend a set of steps, and it requires movement of the damaged knees, hips and ankle, affected by osteonecrosis. This method usually involves children and teenagers in an age range of 4–18 years [6, 10, 34, 35]. TUG is a similar intervention used to evaluate the stage of functional mobility in both healthy children and children with disabilities. In this case it is measured the time needed to stand up from a seated position in a chair, then the patient has to walk for three minutes, turn around, return to the chair and sit down. In this test hand-held dynamometer is also required to measure the level of weakness in these children [36–38].

For the assessment of bone and body composition in patients among 4 to 18 years, Bone Mineral Density (BMD) and Body Mass Index (BMI) are evaluated [39–42]. BMD is searched mainly in the first four lumbar vertebrae, usually the most significant fracture site in children affected by ALL [7, 43–47].

Hartman and collaborators [39] determined BMD in lumbar spine and total body in children affected by ALL. The assessments were performed at diagnosis, during chemotherapy treatment, and one year after the end of treatment. The duration of the intervention was 24 months and involved 51 children, 30 boys and 21 girls. During the 2-year intervention, an exercise program was introduced, consisting of exercises to maintain hand and leg function, stretching exercises to maintain ankle dorsiflexion mobility and short-burst high-intensity exercises (e.g. jumping) to prevent reduction in BMD. In addition, there was a home-based exercise program, consisting of different exercises focused on ankle dorsiflexion mobility, on jumping and stretching, twice daily.

The outcomes revealed a significant positive intervention effect on the total body BMD for the intervention group compared to the control group.

Ness and collaborators [7] conducted an evaluation study on skeletal health, body composition, neuromuscular function, general fitness and self- and parent-reported health-related quality of life (HRQL) among 109 children with a median age of 10 years (range 4–18), newly diagnosed with ALL and compared the results with normative values. Children younger than the age of 4 years were considered not eligible for the analysis. Among the evaluating measures BMD, ankle range of motion, lower extremity strength, motor development, general fitness and BMI were analyzed. The findings revealed that early interventions designed to both preventing bone loss and preserving muscle mass are important for children with newly diagnosed ALL.

Physical therapy interventions for people with ALL are complex because there must be a balance between interventions that promote bone mineral deposition and decreased weight-bearing activities for people with osteonecrosis.

Marchese and collaborators [48] examined physical therapy interventions in more than 20 children (4–15 years old) affected by ALL during chemotherapy. The intervention consisted of 12-week home- and hospital- based intervention program with ankle dorsiflexion stretching exercises (30 seconds, 5 days a week), bilateral lower extremity strengthening exercises (3 sets of 10 repetitions, 3 days a week), and aerobic exercise (daily), such as walking, cycling, or swimming for 4 months. The therapist manually stretched and guided the children to assume specific positions to achieve proper body alignment while performing stretching and strengthening exercises. After 4 months, the children in the intervention group had significantly improved ankle dorsiflexion active range of motion (ROM) and knee extension strength. This suggests that an increased frequency and intensity of intervention on activity is important, as well as a greater emphasis on the child's participation in normal daily activities. The description of the skeletal bone loss impairment observed in children and teenagers with ALL is also reported in Supplementary Table 1.

Prospective longitudinal studies will further determine the efficacy of physical therapy intervention in minimizing deficits in body function/structure, activity, and osteonecrosis in children with ALL.

### Musculoskeletal and neuromuscular system

In addition to the dysfunctions of the bone system and joints, children affected by ALL are at high risk for musculoskeletal and neuromuscular impairments [7, 11, 49, 50]. Reduced hand grip strength, ROM and ankle muscle weakness are attributable also to neuromuscular and musculoskeletal impairments. Neurotoxic agents such as vincristine, dexamethasone and intrathecal methotrexate are reported to worse significantly the whole skeletal and neuromuscular system, and the development of new, molecular targeted drugs is needed [7, 11, 51–53]. Documentation of the onset and development of these impairments during ALL treatments will be important so the timing of appropriate interventions can be determined. Like BMD deficits, distal ROM and strength impairments may be responsive to preventive strategies [54]. Ankle leg and back strength changes can be measured by hand-held dynamometer [55, 56]. Muscle endurance is also evaluated by TUDS test and the 9-minute run-walk test, in which children are instructed to walk or run as far as possible in 9 min with the objective to cover the greatest possible distance [48].

A very recent program, the Stoplight Program (SLP) was developed as a proactive physical therapy intervention directed to promote the maintenance of normal motor skills during chemotherapy in ALL children and teenagers and represents a feasible method to administer a physical treatment (PT) intervention. In this program, each patient received a baseline assessment and a standardized intervention programmed on the basis of the resulted assessments. The Program is aimed to evaluate the ankle dorsiflexion ROM, the ability of transition from lying on the floor to stand up independently, the type of the patient gait and the single leg stance time. Patients could be included in the Program only if they are between 1 and 22 years and have a high-risk ALL. In brief, each patient family receive an age specific handout that contains home exercises which are related to the impairment or to the activity level. A stoplight color explains the level of recommended PT intervention. Children in a red light level receive weekly PT interventions based on stretching, balance, strengthening and gait, children with yellow light usually receive monthly or bimonthly PT services, while children with green level have visits only for evaluations. It has been demonstrated that the participants included in this program improved their motor skills, making the SLP Program a proactive and promising physical therapy intervention [11].

The previously cited physical exercise intervention by Marchese *et al*. [48] showed effects also on musculoskeletal system, since the authors reported that stretching and resistance training improved ankle range of motion and knee extension strength.

In another exercise intervention, back and leg strength were assessed by hand-held dynamometer and general body fitness by TUDS test and the 9-minute run-walk test [56]. After the assessment, exercises that the children (*n* = 40) would be carrying out in the next 3 months were described to the participants. The workout program included active range of motion exercises 5 days a week, 3 times a day, leg muscle exercises for strengtheningthe muscles 3 days a week, 3 times a day and aerobic exercises 3 times a week, once a day, for 30 minutes. The intervention was successful in improving back and leg strength.

Another exercise intervention, during ALL maintenance therapy, was carried out among 7 children of 4–7 years of age [6, 35]. Each session started and ended with a low intensity 15-min warm-up and cool-down period, consisting of cycle ergometer and stretching exercises involving all major muscle groups. The intermediate part of the training session was divided into strength and aerobic exercises, consisting of stretching and running, walking and group games. This program implemented resistance for the major muscle groups and reported muscle strength gain already after eight weeks of training. The description of the musculoskeletal and neuromuscular system impairment observed in children and teenagers with ALL is also reported in Supplementary Table 1.

Children experienced physical and health benefits and felt satisfied after the relatively short-term supervised exercise training program. Other physical programs should include both aerobic and resistance exercise training to induce more significant health benefits with improved functional mobility.

### Cardiovascular and cardiopulmonary impairments

Cardiopulmonary capacity is often impaired in children affected by ALL, during and after treatment [36]. Cardiorespiratory fitness is generally measured on a cycle ergometer or treadmill, with the 9-minute run-walk, the TUDS and TUG tests [35, 48, 55–60]. Cardiopulmonary capacity can be measured by the 20-m shuttle run physical education test, also called the Progressive Aerobic Cardiovascular Endurance Run test (PACER) [61] which measures aerobic fitness and it is so called for the duration of its fulfillment. In general, for the 20m shuttle run test the running pace is imposed by a sound signal, and the initial speed is about 8.5 km/h, and it increases of 0.5 km/h over 1 min intervals. The testing usually finishes when the patient stops, due to fatigue, or when he/she does not reach the line at the same time as the audio signal, on two consecutive occasions [62]. Moreover, there is also a test based on an electronically braked cycle ergometer that is the cardio-pulmonary exercise test (CPET). A study by Sharkey and collaborators [63] examined the effects of a 12-week aerobic training program among childhood ALL survivors who had been treated with anthracycline. Among the 10 patients who completed the twice weekly program, there was an average increase in exercise time CPET of 13 percent (%). They also reported a trend toward improvement in peak oxygen uptake and anaerobic threshold. The previously cited physical exercise interventions by Marchese *et al*. and Tanir *et al*. [48, 56] showed effects also on the cardiovascular system, since the 9-minute run-walk test showed a significant effect depending on the intervention (32 children) compared to usual care (32 children).

Moyer-Mileur and colleagues [55] proposed an intervention which included a 12-month home-based exercise combined with a nutrition program. Children were prescribed to perform a minimum of 3′fifteen-to-twenty-minute' sessions of moderate-to-vigorous activity per week. Moreover, they received nutrition education materials on the basis of the United States Department of Agriculture Food Guide Pyramid and nutrition-related activities monthly. The control group received conventional care. The level of daily activity was measured with a pedometer combined with a diary and a specific questionnaire. After 12 months, regular physical activity and cardiopulmonary fitness were assessed with a pedometer and PACER, respectively. The exercise group recorded more steps on the pedometer and reported better results with PACER than the control group, at the end of the intervention. Therefore, the study revealed that a home-based exercise intervention during maintenance therapy improve cardiovascular system in children with standard-risk ALL and thus encourage greater physical activity. The description of the cardiovascular and cardiopulmonary impairments are also reported in Supplementary Table 1.

### Cancer-related fatigue (CRF)

Cancer-related fatigue (CFR) represents one of the most serious and frequent symptoms observed in pediatric patients affected by ALL, during the cycles of chemotherapy but also after the end of the treatments [64–68]. It usually reaches high intensity in the first few days after chemotherapy and then gradually decreases until the next course of pharmacological treatments [69]. Fatigue involves the whole physical and psychosocial aspects and also impacts future planning and child self-performance [70]. Non-pharmacological management, including counseling and education, or pharmacological interventions (for pain, sleep medication or treatment of anemia) are general strategies for the management of fatigue [64]. Literature data report that physical exercise could exert more effective benefits than other approaches such as sleep promotion, relaxation or distraction, in attenuating fatigue [71–73]. Most of the training program includes three to five training sessions per week, with a range from nearly 3 weeks to 4–6 months and in most programs the duration of sessions is gradually increased until continuous exercise for about 30 minutes was achieved [74]. At the end of an exercise intervention program, fatigue can be determined by a validated Checklist Individual Strength (CIS-20) questionnaire [75, 76], where the patient is asked to measure four aspects about fatigue in the 2 weeks preceding the evaluation: subjective experience of fatigue, concentration, motivation, and physical activity.

Yeh and colleagues [69] proposed a 6-week home-based aerobic exercise intervention to reduce fatigue in ALL children during maintenance chemotherapy. The content of the home-based intervention is composed of 3 different sections: a warm-up phase of 5 minutes, 25 minutes of aerobic exercise and 5 minutes of cooling down. Fatigue was measured according to the Teenagers Pediatric Quality of Life PedsQL Multidimensional fatigue scale [77–79], which classifies the fatigue into general, sleep/rest fatigue and cognitive fatigue (6 items each) [80, 81], ranging from 1= “not at all” to 5= “always”. The study was conducted on 22 children, the majority of them included in the intervention group. Children who received exercise intervention showed lower general fatigue than those in the control group at the 1-month follow-up measurement suggesting thus that a home-based exercise program can significantly reduce fatigue during ALL maintenance therapy. Of note, family involvement with structured activities is an effective way to increase physical activity among children with ALL [64]. Hooke *et al*. [82] explored if children who received daily a fitness tracker (FitBit^®^) coaching for 2 weeks before a maintenance steroid pulse had an increase in steps per day, and determined the relationship between steps per day prepulse and fatigue postpulse. FitBit^®^ is a sensor that clips to clothes, measures steps, motion assessment of fatigue, and uploads the data to a website when the sensor is within range of the base station. Seventeen children in ALL maintenance, aged 6–15, wore the FitBit^®^ continuously starting on day −17 before a corticosteroid pulse and continue wearing it through day 5 of the corticosteroid pulse. FitBit^®^ provided feedback on walking goals to the participant as well as measuring the steps taken per day. Daily emails with comments and FitBit^®^ screenshots were sent over the 2-week intervention, and fatigue was measured after 2 weeks. Results reported that the average steps of each time period (week 1, week 2, and during steroid treatment) was very high, reporting that ALL children can be active also during treatment. Moreover, higher steps were associated with lower fatigue, strengthening the concept that physical activity is protective for fatigue during a corticosteroid pulse.

Physical exercise aimed to improve fatigue is expected to be more successful and time-efficient in children with fatigue in ALL rather than in adult patients, and this is correlated to the fact that in children there is no chronic deconditioning before the disease, usually present in adult cancer patients and in the elderly. Secondly, children have more adaptability to training and their body tissues are more elastic and more flexible.

Exercise physiologists can at any time assist oncologists in prescribing exercise programs for attenuating cancer-related fatigue and therefore ensuring a general improvement for ALL children.

The description of the fatigue impairment observed in children and teenagers with ALL is also reported in Supplementary Table 1.

### Balance impairment

Balance, also known as postural stability, is defined as the ability to control one's center of mass within the base of support. In order to maintain balance, any person needs information from three different sensory systems: visual, somatosensory, and vestibular. Postural control, the ability to control the position and orientation of the body in space is a requirement for maintaining balance during different activities [83, 84]. Information about balance is an important component of the assessment of motor skills in children, primarily because of its impact on functional abilities such as play, recreation and activities of daily living. Problems with balance as well as limitations in running speed and strength have been identified following treatment for ALL using standardized gross motor assessments. Treatments of acute lymphoblastic leukemia with chemotherapeutic agents such as vincristine can damage balance via different mechanisms, including sensory and motor peripheral neuropathy, cognitive impairment and reduced muscle strength and flexibility. Children with ALL, because of their potential somatosensory, motor, muscular and cognitive impairments, are more likely to experience balance problems [83] with particular severity if those impairments are diagnosed before the age of 4 years, which is a critical time window in the development of postural control. Disorders in postural control can preclude children from doing several activities in daily life, as well as sports activities. For this reason, identifying balance disorders in children at recent diagnosis may help to prevent additional motor balance problems, increase physical training performances and team activities, and in general improve the quality of life. With this aim, the validation of promising balance tests is absolutely necessary.

A relevant test for measuring balance is the Movement Assessment Battery for Children (ABC) [85]. The assessment gives quantitative and qualitative data about a child's performance of age-appropriate tasks within 3 subsections: Manual Dexterity, Ball Skills, and Static and Dynamic Balance. The Movement ABC is a task set designed to screen for motor impairment rather than provide a profile of a child's motor performance. It takes approximately 30 minutes and requires no special training.

Galea and collaborators [86] proposed different tests for ALL patients, in which six different conditions were assessed taking a basis on exercises with opened or closed eyes. All these tests were promising, although some patients were unable to complete the most challenging task, in which they were required to balance over a narrow base of support with closed eyes. Similarly, Leone *et al* [87] assessed balance with two tests based on exercises with opened or closed eyes. These tests were included in a wider test Program called Gross Motor Skills (GMS) Test, that also included agility, coordination, reaction time and limb speed tests. The GMS Test appeared to be a useful tool to quantify the extent of the ALL children motor impairment.

It was found a negative association between body mass index and balance [54, 88, 89], and it was seen that vincristine treatment has a negative influence on balance performance [88]. In a recent work, ALL survivors of more than 10 years old participated in a neuromuscular performance testing in which ankle tendon reflexes, touch and vibratory sensation, ankle range of motion and strength, quadriceps strength, balance, mobility and walking efficiency were evaluated. Balance was assessed by completing the sensory organization test (SOT) on a computerized dynamic posturography system, in which participants were asked to stand on dual force plates that were able to provoke ankle motion [54]. It was seen that higher cumulative doses of vincristine and/or intrathecal methotrexate were associated with neuromuscular and balance impairments which have implications on future function as these survivors age.

Finally, it seems to exist a relationship between cognition and balance [90] that raises questions about the impact of these factors on a child's quality of life. While the causative nature of this relationship is unclear, activities that involve both cognition and balance concurrently are likely to be more challenging for survivors and potentially raise safety concerns. For example, texting while walking may be more difficult for a survivor and place the individual at higher risk of falling.

A correct balance is essential for children, and it is also very important to delineate a correct posture. Therefore, additional studies that could give a more detailed explanation of changes in balance both during and after the completion of treatment will be of help.

The description of the balance impairment is also reported in Supplementary Table 1.

### Obesity and metabolism disorders in ALL survivors

Childhood leukemia has an increasing number of survivors; therefore more emphasis is focused on the long-term effects of this disease and on the pharmacological treatments with minor toxic effects [91–93]. As reported previously, the chemotherapeutic treatment in ALL children can give short and long term effects and alterations. In particular, it was observed that after a corticosteroid therapy, especially with dexamethasone and prednisone, some ALL survivors are obese or accuse metabolism disorders [93]. Warner and collaborators [94] reported for ALL patients a strong negative relationship between exercise capacity, including sub-maximal oxygen consumption, and adiposity. This is in part due to a reduction of accumulation of mitochondria in ALL muscle patients. The result is a reduced oxygen uptake during sub-maximal exercise as well as the reduced capacity in leukemia patients to oxidize fats as a fuel. In addition to obesity, hyperlipidemia, dyslipidemia and diabetes mellitus are other significant late effects among ALL children. Many children are obese at the time of diagnosis, and excessive weight gain occurs early in therapy, regardless of age, sex or degree of obesity at the time of diagnosis [95].

The mainstay of intervention for obesity and metabolic disease in the general population is diet and exercise [96]. A study by Jarvela *et al.* assessed the efficacy on obesity and metabolism disorders of a 16-week, home-based exercise program for long-term survivors of ALL [97]. The ALL survivors involved in the study were between the ages of 16 and 30, with all participants at more than 10 years from diagnosis. Peak oxygen uptake (VO2 peak), muscle strength, and metabolic risk factors were studied before and after the exercise program. The muscle training program consisted of eight exercises to strengthen gluteal and lower limb muscles, shoulders and upper limb, abdominal and back muscles. In the study several risk factors were evaluated: fasting insulin, homeostasis model assessment, insulin resistance (HOMA-IR), fasting glucose, lipid profiles, and systolic blood pressure. It was seen that home based exercise program induced a significant improvement in fasting insulin and systolic blood pressure at the end of the study period. Additionally, there was a significant decrease in waist circumference, waist-to-hip ratio, and fat percentage.

Adiposity, usually expressed as an increased BMI, is associated with adverse levels of the cardiovascular risk factors and insulin resistance in pediatric populations [98, 99]. However, in this population BMI provided a less accurate representation of adiposity, which was identified only after dual-energy X-ray absorptiometry (DXA) screening. It is reasonable to consider that in the presence of decreased insulin sensitivity and adverse levels of cardiovascular risk factors, childhood cancer survivors have a higher potential for the development of early cardiovascular impairments than controls, as they progress into adulthood. Early development of altered body composition and decreased insulin sensitivity in survivors may contribute significantly to their risk of early cardiovascular morbidity and mortality.

Because of growing interest in the effectiveness of diet and exercise on cardiometabolic risk modification in the childhood cancer population, a number of National Institutes of Health–supported studies are looking to provide insight into effective interventions. For example, Let's Play! Healthy Kids After Cancer is a nutrition and physical activity intervention targeted to parents of young ALL survivors (age 4–10 years old). The study aims to modify the home environment to improve physical activity and diet, so as to mitigate unhealthy weight gain after chemotherapy. At the end of the 6-month intervention period, changes in weight-related behaviors have been noted, as well as in biomarkers of inflammation and oxidative stress, fatigue, and body composition [96].

The description of the Obesity and Metabolism disorders is also reported in Supplementary Table 1.

Thus far, exercise intervention studies in childhood cancer survivors have shown improvements in different metabolic aspects. Concerning metabolism disorders, the ongoing studies have to further develop the appropriate diet and exercise prescription for the at-risk childhood cancer survivors. Investigation of the mechanism of metabolic diseases may help to modify therapeutic regimens during cancer treatment itself. Further research is warranted to evaluate the barriers to a healthy lifestyle and other interventions in childhood cancer survivors.

## CONCLUSIONS

It has been shown that physical exercise has beneficial effects during pharmacological treatments of ALL children and survivors. The published evidence is positive for the impact of exercise on skeletal bone, musculoskeletal and neuromuscular systems, cardiopulmonary and cardiovascular aspects, body balance and metabolism disorders. Moreover, there is scientific evidence that exercise can attenuate cancer-related fatigue and improve the wellbeing of ALL patients and survivors. During ALL course these elements are strongly altered, thus inducing a worsening of the general child quality of life and physical weakness associated with a progressive worsening of the physical and psychological aspect. The emphasis on ALL children in participating in physical activities is noteworthy, because ALL pediatric patients can often show some depressive symptoms such as negative mood, interpersonal problems, ineffectiveness, anhedonia, and negative selfesteem. Physical exercise training, as well as game activities, can strengthen a relationship of trust and security among the subjects involved, ameliorating the child psychological aspect, reinforcing the importance of the interpersonal component and generally improving the quality of ALL children life [16].

To date, recent physical exercise interventions are available for ameliorating bone loss impairment (from 4 month to 2 year interventions), musculoskeletal impairment (from 8 week to 3 month interventions), cardiovascular and cardiopulmonary systems (from 12 week to 12 month physical programs), cancer-related fatigue, with most of the training interventions ranging from 3 weeks to 4–6 months, and metabolism disorders with actually two program interventions, one of 16-weeks and the second one of 6-months. Identifying balance disorders in children at recent diagnosis is necessary to prevent additional motor balance problems, increase physical training performances and team activities, and in general improve the quality of life.

The results obtained from the above described physical exercise interventions are promising, and further researches will highlight the importance of physical activity in improvement of ALL impairments. Studies designed to identify and characterize the type and intensity of exercise useful to achieve clinically meaningful outcomes on skeletal, musculoskeletal, neuromuscular, cardiopulmonary and cardiovascular systems, fatigue, body balance disorders, metabolism alterations and quality of life in ALL children are ongoing. These interventions must be realistic and portable so that ALL children and their families can adopt and incorporate them in daily life exercise and physical activity, especially when they are not near the specialized center that provides care and services for children with cancer.

## SUPPLEMENTARY MATERIALS TABLES




