# Supplementary material for: Physical training interventions for children and teenagers affected by acute lymphoblastic leukemia and related treatment impairments
Source: Oncotarget. 2018 Mar 30;9(24):17199–209. doi: 10.18632/oncotarget.24762 (PMC5908317; doi:10.18632/oncotarget.24762)
Supplement: Supplementary file 1 [file oncotarget-09-17199-s001.pdf]

# Physical training interventions for children and teenagers affected by acute lymphoblastic leukemia and related treatment impairments

## SUPPLEMENTARY MATERIALS

**Supplementary Table 1: Description of the common impairments observed in children and teenager with ALL, the measures and the tests used for their evaluation, and the proposed physical exercise intervention programs.** See Supplementary_Table_1
