# Supplementary material for: Physical training interventions for children and teenagers affected by acute lymphoblastic leukemia and related treatment impairments
Source: Oncotarget. 2018 Mar 30;9(24):17199–209. doi: 10.18632/oncotarget.24762 (PMC5908317; doi:10.18632/oncotarget.24762)
Supplement: Supplementary file 2 [file oncotarget-09-17199-s002.docx]

**Supplementary Table 1: Description of the common impairments observed in children and teenager with ALL, the measures and the tests used for their evaluation, and the proposed physical exercise intervention programs**

| **Impairment** | **Measure Tests and Methods** | **Physical Exercise Intervention Program**  **And References** | **Obtained Results** |
| --- | --- | --- | --- |
| **Skeletal bone system:**  -Bone loss  -Decreased Bone Mineral Density (BMD)  -Bone demineralization  -Suppression of osteoblastic differentiation  - Osteonecrosis | -Magnetic resonance imaging (MRI)  -Size of necrotic area: Association Research Circulation Osseus (ARCO)  -Dual X-ray absorptiometry (DXA)  **-**Timed up and down stairs (TUDS)  -Timed Up and Go (TUG) | 1) **4-month intervention:**-exercises focused on ankle dorsiflexion stretching  (5 days a week)  -exercises focused onlower-extremity strengthening  (3 days a week)  -aerobic exercise: daily REF **[48]**  2) **2-year intervention:**  -exercises for hand and leg function;  -stretching exercises for ankle dorsiflexion mobility and short-burst high-intensity exercises;  -home-based exercises program: exercises focused on ankle dorsiflexion mobility, jumping and stretching twice daily.  REF **[39]** | 1) Improvement of ankle dorsiflexion active range of motion (ROM) and knee extension strength.  2) Significantpositive intervention effect on the total body BMD for the intervention group compared to the control group. |
| **Musculoskeletaland neuromuscular system:**  -Changes inAnkle Range of Motion (ROM)  -Changes inankle leg and back strengths  - Changes inmuscle endurance  -Lower extremity Strength  -Motor development  - Decreased Body Mass Index (BMI) | **-**Timed up and down stairs (TUDS)  -The 9-minute run-walk test  -Hand-held dynamometer | 1) **The Stoplight Program:**  Evaluation of ankle dorsiflexion ROM, the ability to transition from lying on the floor to stand up independently, the type of the patient gait and the single leg stance time.  REF **[11]**  2) **4-month intervention:**--exercises focused on ankle dorsiflexion stretching  (5 days a week)  -exercises focused onlower-extremity strengthening  (3 days a week)  -aerobic exercise: daily REF **[48]**  3) **3-month intervention:**  - active range of motion (5 days a week, 3 times a day) -leg muscle strengthening (3 days a week, 3 times a day)  -aerobic exercises (3 times a week, once a day, for 30 minutes).  REF **[56]**  4) **8-week training intervention:**  -15-min warm-up and cool-down period: cycle ergometer and stretching exercises;  -strength and aerobic exercises: stretching and running, walking and group games.  REF **[6, 35]** | 1)Improvement of motor skills.  2) Improvement of ankle range of motion and knee extension strength.  3) Improvement of the back and leg strength.  4) Implementation resistance for the major muscle groups and muscle strength gain |
| **Cardiovascular and Cardiopulmonary systems:**  - Alterations in in peak oxygen uptake and anaerobic threshold | **-**Progressive Aerobic Cardiovascular Endurance Run test (PACER)  -Cardio-pulmonary exercise test (CPET)  -Use of a cycle ergometer or treadmill  -The 9-minute run-walk test  -TUDS and TUG test  - pedometer | 1) **12-week intervention:**  aerobic training with home  exercise twice per week.  REF **[63]**  2) **4-month intervention:**  -exercises focused on ankle dorsiflexion stretching  (5 days a week)  - exercises focused on lower-extremity strengthening  (3 days a week)  -aerobic exercise: daily REF **[48]**  3) **3-month intervention:**  - active range of motion (5 days a week, 3 times a day) -leg muscle strengthening (3 days a week, 3 times a day)  -aerobic exercises (3 times a week, once a day, for 30 minutes).  REF **[56]**  4) **12-month intervention:** -home-based exercises combined with a nutrition program: 3 ’fifteen to twenty-minute’ sessions of moderate-to-vigorous activity per week.  REF **[55]** | 1) Improvement in peak oxygen uptake and anaerobic threshold  2) Significant improvement on cardiovascular system in general  3) Significant improvement on cardiovascular system in general  4) Significant improvement on cardiovascular system in general |
| **Cancer-related fatigue**  -Whole body tiredness | -Teen Pediatric Quality of Life PedsQL Multidimensional fatigue scale  -validated Checklist Individual Strength (CIS-20) questionnaire  -counseling and education  -sleep promotion  -relaxation or distraction  -FitBit® fitness tracker | 1) **Most of the training interventions:**  three to five training sessions per week, 3weeks to 4-6 months  REF **[74]**  2) **6-week home-based aerobic exercise intervention**:  -a warm-up phase of 5 minutes  -25 minutes of aerobic exercise  -5 minutes of cooling down  REF **[64]**  3) **2-week intervention:**  walking  REF **[82]** | 1) Reduction of fatigue  2) Reduction of fatigue during ALL maintenance therapy.  3) Reduction of fatigue |
| **Balance:**  -Postural control and coordination weakness | -Movement ABC  -Sensory Organization Test (SOT)  -Gross Motor Skills (GMS) Test | 1) Interventions based on exercises with open or closed eyes  REF **[86, 87]** | 1) Improvement of balance |
| **Metabolism Disorders:**  -Obesity  -hyperlipidemia  -dyslipidemia  -diabetes mellitus | -Dual X-ray absorptiometry (DXA) | 1) **16-week home-based intervention:**  **-** exercise program for long-term survivors: focus on strength of lower limb muscles, shoulders and upper limb, abdominal and back muscles  REF **[97]**  2) **6-months Let's Play! Healthy Kids After Cancer intervention:**  **-**modification of the home environment to improve physical activity and diet of young ALL survivors.  REF **[96]** | 1) A significant improvement in fasting insulin and systolic blood pressure at the end of the study period  -A significant decrease in waist circumference, waist-to-hip ratio, and fat percentage  2) Changes in weight-related behaviors, as well as in biomarkers of inflammation and oxidative stress, fatigue, and body composition |
